# Neuroprotective Potential of Synthetic Mono-Carbonyl Curcumin Analogs Assessed by Molecular Docking Studies

**DOI:** 10.3390/molecules26237168

**Published:** 2021-11-26

**Authors:** Haya Hussain, Shujaat Ahmad, Syed Wadood Ali Shah, Mehreen Ghias, Abid Ullah, Shafiq Ur Rahman, Zul Kamal, Farman Ali Khan, Nasir Mehmood Khan, Juma Muhammad, Mazen Almehmadi, Osama Abdulaziz, Saad Alghamdi

**Affiliations:** 1 Department of Pharmacy, Shaheed Benazir Bhutto University Sheringal, Upper Dir 18000, KPK, Pakistan; haya@sbbu.edu.pk (H.H.); abid@sbbu.edu.pk (A.U.); shafiq@sbbu.edu.pk (S.U.R.); xulkamal@sbbu.edu.pk (Z.K.); 2 Department of Pharmacy, University of Malakand Dir (Lower), Chakdara 18800, KPK, Pakistan; mehreenghias@yahoo.com; 3 Department of Chemistry, Shaheed Benazir Bhutto University Sheringal, Upper Dir 18000, KPK, Pakistan; farmanali@sbbu.edu.pk; 4 Department of Agriculture, Shaheed Benazir Bhutto University Sheringal, Upper Dir 18000, KPK, Pakistan; nasir@sbbu.edu.pk; 5 Department of Environmental Sciences, Shaheed Benazir Bhutto University Sheringal, Upper Dir 18000, KPK, Pakistan; juma@sbbu.edu.pk; 6 Clinical Laboratory Sciences Department, College of Applied Medical Sciences, Taif University, Taif 26513, Saudi Arabia; dr.mazen.ma@gmail.com (M.A.); o.osama@tu.edu.sa (O.A.); 7 Laboratory Medicine Department, Faculty of Applied Medical Sciences, Umm Al-Qura University, Makkah 24211, Saudi Arabia; ssalghamdi@uqu.edu.sa

**Keywords:** mono-carbonyl curcumin analogs, cholinesterases, molecular docking, amnesia, scopolamine, EPM, NORT, hippocampus

## Abstract

Cognitive decline in dementia is associated with deficiency of the cholinergic system. In this study, five mono-carbonyl curcumin analogs were synthesized, and on the basis of their promising in vitro anticholinesterase activities, they were further investigated for in vivo neuroprotective and memory enhancing effects in scopolamine-induced amnesia using elevated plus maze (EPM) and novel object recognition (NOR) behavioral mice models. The effects of the synthesized compounds on the cholinergic system involvement in the brain hippocampus and their binding mode in the active site of cholinesterases were also determined. Compound **h2** (*p <* 0.001) and **h3** (*p <* 0.001) significantly inhibited the cholinesterases and reversed the effects of scopolamine by significantly reducing TLT (*p <* 0.001) in EPM, while (*p <* 0.001) increased the time exploring the novel object. The % discrimination index (DI) was significantly increased (*p <* 0.001) in the novel object recognition test. The mechanism of cholinesterase inhibition was further validated through molecular docking study using MOE software. The results obtained from the in vitro, in vivo and ex vivo studies showed that the synthesized curcumin analogs exhibited significantly higher memory-enhancing potential, and **h3** could be an effective neuroprotective agent. However, more study is suggested to explore its exact mechanism of action.

## 1. Introduction

Dementia is related to cognitive decline in brain function, and Alzheimer’s disease (AD) is the most common form of dementia in elderly people, characterized by cognitive decline associated with memory loss, impaired judgment, reasoning and communication; it is a globally growing health challenge [1]. The literature shows that AD has been reported in more than 35 million people globally, and after diagnosis leads to death within 3 to 9 years [2,3]. The pathogenesis of dementia is illustrated by the cholinergic hypothesis [4], in which neuronal damage in the septohippocampal cholinergic system progresses the disease [5], leading to loss of memory and cognition [6]. Short-term memory loss is the distinguishing feature of the disease, associated with minor forgetfulness, and after chronic cascade, it leads to death [7]. Sensations, impressions, and ideas are preserved and recalled in the elementary mental process of memory [8]. Learning is the acquisition of information and skills, while their subsequent retention is called memory, and cognition is collectively known for learning and memory [9]. Although Alzheimer’s disease remains the focus of enormous amounts of experimental work, yet the exact localization of neuropathological changes essential for memory loss and the underlying cognitive mechanisms remain unexplored [10]. 

Acetylcholine (ACh) is the most important neurotransmitter responsible for maintaining cognitive functions. The cholinergic system plays an important role in cognitive symptoms in dementia, and the cholinergic hypothesis illustrates that disturbances in cholinergic activity in the brain are responsible for decline in cognition and memory loss [11]. The cholinergic system in the basal forebrain is responsible for the processes of cognition and memory [12,13]. Cholinergic neurotransmission plays a vital role in encoding memory and attention [13]. The centrally acting cholinergic system retains memory by maintaining ACh levels [14], and low levels result in cognitive impairment, leading to memory loss, whcih can be cured by the administration of cholinesterase inhibitors [15].

Acetyl cholinesterase (AChE) inhibitors have been explored so far to relieve symptoms of dementia by activating the cholinergic system. Galantamine, donepezil, rivastigmine and tacrine are cholinesterase inhibitors that have been approved for symptomatic aid in dementia in AD [16,17,18].

Curcumin is naturally found in *Curcuma longa L* and has traditionally been used as a dietary pigment, and has been extensively used as an antioxidant, anti-inflammatory and antitumor agent [19,20,21]. Nowadays, the role of curcumin in treating neurodegenerative diseases, including Alzheimer’s disease (AD) and Parkinson’s disease (PD), is attracting significant interest [22]. It has been reported in many studies that curcuminoids have memory enhancing potential in experimental animal models [23], and that the memory enhancing effect may be due to curcumin [24]. Several researchers have reported that synthetic molecules can enhance the ability of learning and memory, thereby acting on the central nervous system [6].

Bearing in mind the aforementioned properties of curcumin, the current study was conducted. A series of synthetic mono-carbonyl curcumin analogs were synthesized and evaluated for their possible neuroprotective potential to introduce alternative therapeutic molecules for AD. 

## 2. Results

### 2.1. Chemistry

**Figure 1:**
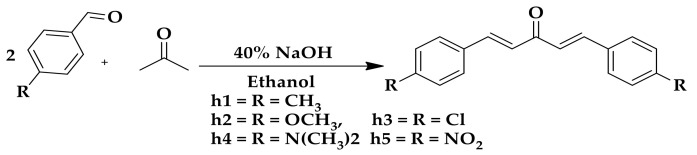
Mono-carbonyl curcumin analogs used in this study for memory enhancing effect.

A series of five mono-carbonyl curcumin analogs (**h1**–**h5**) possessing substituted aromatic rings with various functional groups were synthesized from acetone in the presence of ethanol at room temperature (Figure 1).

### 2.2. Molecular Docking Study

The synthesized compounds were docked using the MOE-Dock protocol to predict their interaction with the targets AChE and BChE, as well as ligand molecules; the ligX application within the MOE package was also used [25]. The MOE tool was used, and all compounds showed good binding affinity with the target proteins. The binding modes of compounds were in correlation with the calculated IC_50_ values.

#### Molecular Docking Validation of Synthesized Compounds for Anticholinesterases 

**Figure 2:**
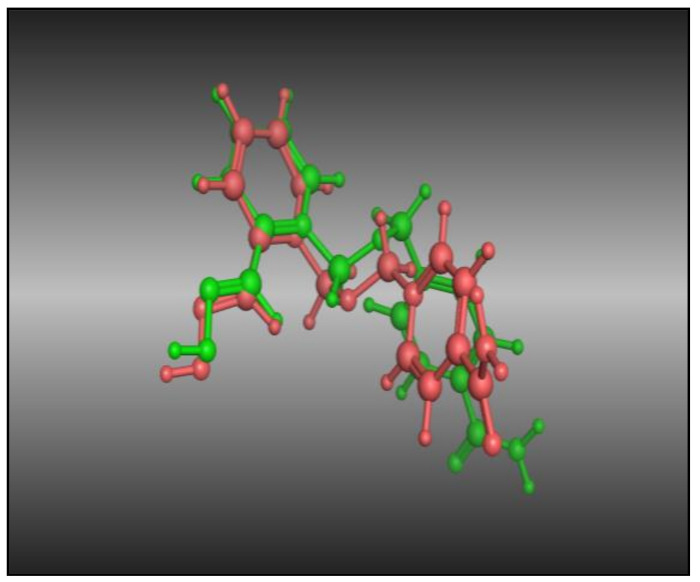
Dark pink: co-crystallized ligand; green: dock ligand.

The co-crystallized ligand was re-docked in the acetyl cholinesterase (PDB ID: 2gyu) inhibitor binding cavity after removing the co-crystallized ligand from the active sites. An RMSD value of 1.618 Å was observed (Figure 2), indicating that the incorporated docking protocol was valid for this research work, and that the MOE-Dock method was reliable for the docking study of these synthesized curcumin analogs.

**Figure 3:**
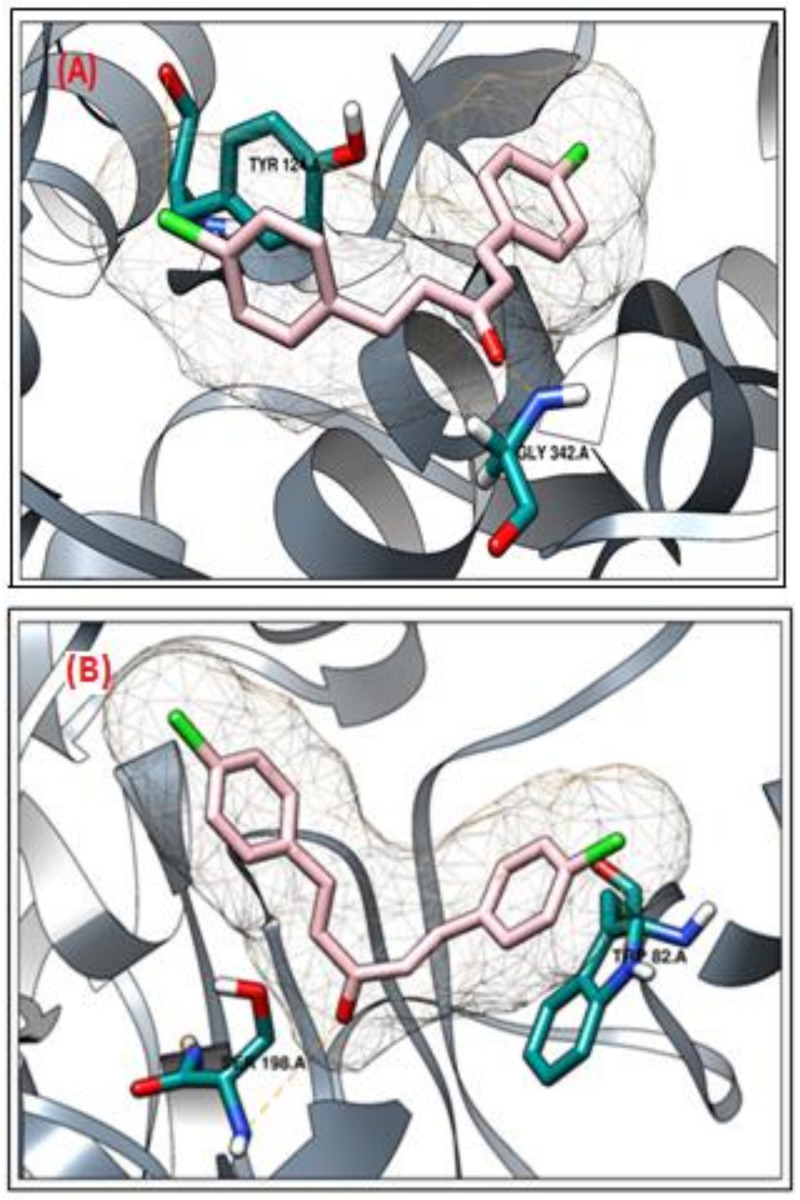
3D binding mode of compound **h3** against AChE (**A**) and BChE (**B**) proteins.

In the case of acetyl cholinesterase (AChE), the active compound **h3** showed the most promising results. The compound **h3** interacted with two active site residues (Tyr124 and Gly342). The active site residue Tyr124 interacted with the chlorobenzene moiety, and Gly342 interacted with the carbonyl group of the compound (Figure 3A). Similarly, as observed in the case of AChE, the compound **h3** was the most active against the butyryl cholinesterase enzyme (BChE). The compound **h3** interacted with two active site residues (Trp82 and Ser198). The active site residue Trp82 interacted with the chlorobenzene moiety and Ser198 interacted with the carbonyl group of the compound (Figure 3B). 

Table 1 shows the binding energies and docking scores for the targets docked with the synthesized compounds. The analysis of the binding interactions of the synthesized compounds demonstrated that the compounds were precisely docked into the active site residues and showed promising results against both enzymes.

### 2.3. Pharmacological Activities 

#### 2.3.1. In Vitro Cholinesterase Inhibition Potential of Curcumin Analogs

Cholinesterase inhibition potential was determined for the tested compounds against AChE and BChE enzymes in concentrations of 1000 to 62.5 μg/mL, as shown in Table 2. Compound **h3** showed significantly higher inhibition potential against AChE, with an IC_50_ value of 29.39 μg/mL, followed by **h2**, with an IC_50_ value of 93.27, and **h1**, with an IC_50_ 104.72 μg/mL. Compound **h4**, with an IC_50_ of 294.77, and **h5**, with an IC_50_ of 632.37 μg/mL showed weak inhibition potential and galanthamine was used as standard control with an IC_50_ of 26.58 μg/mL. Similarly, the synthesized curcumin analogs were tested against BChE in the same concentrations as AChE and the results showed that compound **h3** exhibited significantly higher inhibition potential, with an IC_50_ of 67.35 μg/mL, followed by **h2**, with an IC_50_ of 147.92, and **h1**, with an IC_50_ of 243.12 μg/mL, compound **h4**, with and IC_50_ of 512.77, and **h5**, with an IC_50_ of 988.35 μg/mL, as shown in Figure 3. 

#### 2.3.2. In Vivo Study

##### Elevated Plus Maze

The results of the EPM mice model for the synthesized curcumin analogs are shown in Table 3. Thirty minutes after the last dose on day 7, there was marked memory impairment (amnesia) produced upon administration of 1 mg/kg i.p. scopolamine, and a higher transfer latency time (TLT) was demonstrated compared to the normal control group. Administration of the standard drug donepezil significantly reduced the retention time in seconds, with a TLT of 25.57 ± 1.93 s (*p <* 0.001), compared to the amnesic group. Similarly, synthesized curcumin analogs were administered at doses of 7.5 and 15 mg/kg p.o. and treatment was administered for 7 days, demonstrating a significant reduction of TLT compared to the amnesic group after scopolamine administration. Compound **h3** exhibited a significant reduction, with a TLT of 25.26 ± 2.51 s (*p <* 0.001) at 7.5 mg/kg and 21.27 ± 2.92 s (*p <* 0.001) at 15 mg/kg, indicating a reversal of cognitive decline and enhanced memory. Compound **h2** exhibited a reduction, with a TLT of 31.51 ± 2.73 s (*p <* 0.01) and 25.32 ± 2.42 s (*p <* 0.001), respectively, at the two doses. Similarly, **h1** exhibited a significant reduction, with a TLT of 26.18 ± 2.32 s (*p <* 0.001) at 15 mg/kg compared to the amnesic group. Compound **h4** exhibited a reduction, with TLT values of 35.71 ± 1.13 s (*p <* 0.05) at 7.5 mg/kg and 30.42 ± 1.24 s (*p <* 0.01) at 15 mg/kg, while **h5** exhibited a reduction, with a TLT of 34.71 ± 1.37 s (*p <* 0.05) and 31.51 ± 1.54 s (*p <* 0.01) in comparison with amnesic group at the two doses, respectively.

##### Novel Object Recognition Test

**Figure 4:**
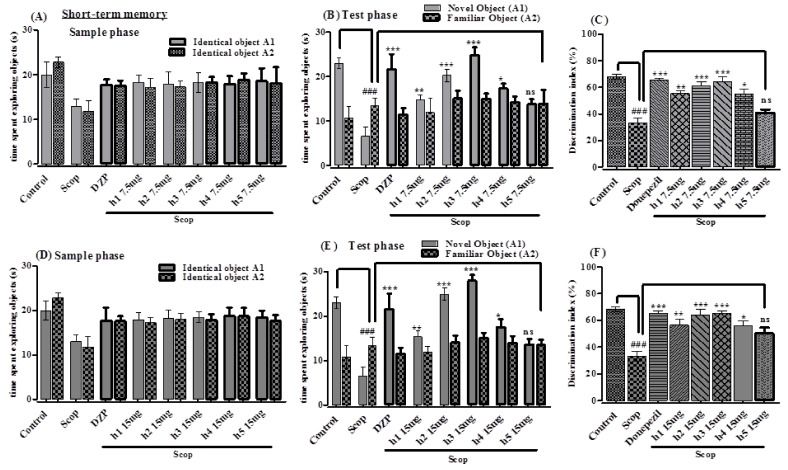

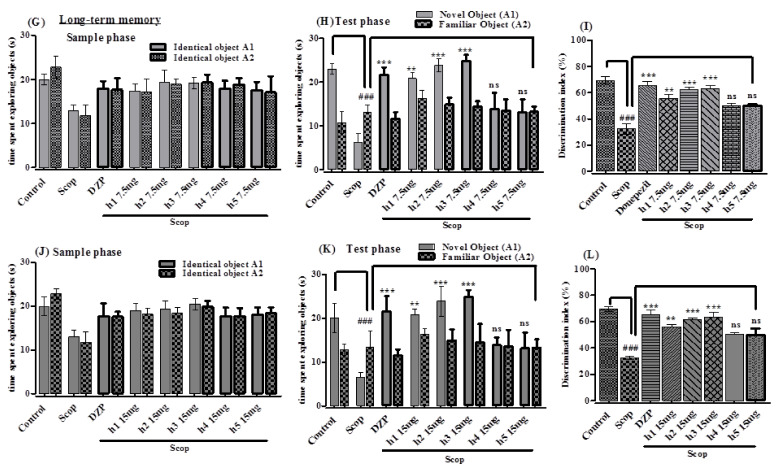
The effect of the synthesized curcumin analogs (h1–h5) in NORT (**A**,**D**,**G**,**J**) sample phase, (**B**,**E**,**H**,**K**) test phase (**C**,**F**,**I**,**L**) % DI versus scopolamine-treated group for the evaluation of short- and long-term memory. All values are presented in mean ± SEM (*n* = 6), ### *p* < 0.001 vs. normal control. Significantly different values are with respect to the amnesic (scopolamine) group; *p*-value <0.001, <0.01, <0.05, >0.05 are expressed as ***, **, * and ‘ns’, respectively. One-way ANOVA followed by Dunnett’s multiple comparison tests was applied on this data.

The synthesized compounds were investigated for short-term memory in scopolamine-induced memory impairment in mice using the NOR test. There was no significant change in the sample phase observed in assessing short-term memory in time consumed for exploration of both identical objects between treatment with any of the samples and the scopolamine-treated group (Figure 4A,D). There were significantly higher exploration times for the novel object than for the identical object recorded in the test phase when treated with **h1**–**h5** at 7.5 and 15 mg/kg and DZP 2 mg/kg. The administration of the standard drug donepezil significantly increased the exploration time in seconds to 21.59 ± 2.44 (*p <* 0.001) for the novel object, with a discrimination index (DI) of 65.26%, and decreased it for the familiar object to 11.49 ± 1.39, compared with the amnesic group (scopolamine). Animals in the scopolamine-treated group spent more time with the familiar object compared to the novel object, having a significant % DI (*p <* 0.001) in the test phase. Animals treated with **h2** and **h3** spent significantly more time exploring the novel object in the test phase compared to the scopolamine-treated group (Figure 4B,E). The % DI values for **h2** (*p <* 0.001) and **h3** (*p <* 0.001) were significant, while **h1** (*p <* 0.01) and **h4** (*p <* 0.05) showed comparable values at 7.5 and 15 mg/kg (Figure 4C,F). Compound **h5** showed no promising results in this study (*p >* 0.05). Similarly, when assessing long-term memory, there was no significant change found in the time taken for exploring both of the identical objects in the sample phase between any of the samples and the scopolamine-treated groups (Figure 4G,J). There was a significant difference observed in the test phase with increased time exploring the novel object between the groups administered with the samples (7.5 and 15 mg/kg) and the group administered with standard donepezil (2 mg/kg). The amnesic (scopolamine-treated) group spent more time (*p <* 0.001) exploring familiar object compared to the novel object (Figure 4H,K). The standard drug donezepil (*p <* 0.001) (2 mg/kg) and the compounds **h2** (*p <* 0.001), **h3** (*p <* 0.001), **h1** (*p <* 0.01) showed a significant increase in the % DI in exploring the novel object at 7.5 and 15 mg/kg doses. Compounds **h4** and **h5** showed no promising results (*p >* 0.05) with respect to % DI. The amnesic (scopolamine-treated) group exhibited a significantly (*p <* 0.001) lower % DI in comparison with the control group (Figure 4I,L).

#### 2.3.3. Effect of Synthesized Curcumin Analogs on Brain Hippocampus Cholinesterase (AChE and BChE) Levels

**Figure 5:**
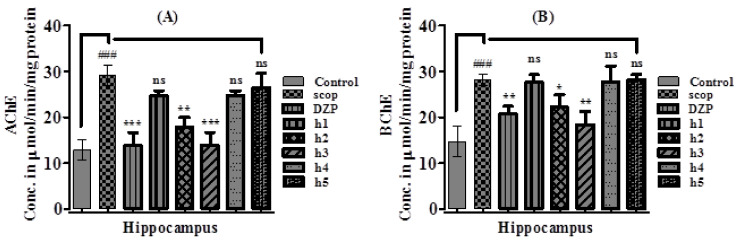
Ex vivo anticholinesterase effect of synthesized curcumin analogs (**h1**–**h5**) in AChE (**A**) and BChE (**B**) in the hippocampus region of mouse brain versus the scopolamine-treated group. All values are presented as mean ± SEM (*n* = 6), ### *p* < 0.001 vs. normal control. Significantly different values are with reference to the scopolamine-treated group; *p*-value <0.001, <0.01, <0.05 are expressed as ***, **, and *, respectively. One-way ANOVA followed by Dunnett’s multiple comparison tests was applied.

This study showed that upon administration of scopolamine, the AChE level was substantially increased to 29.17 ± 2.14 µmol/min/mg of protein (*p* < 0.001, *n* = 6) in the hippocampus compared to 12.83 ± 2.23 µmol/min/mg of protein in the control group, which was reversed by the pretreatment with donepezil 13.91 ± 2.82 µmol/min/mg of protein (*p* < 0.001, *n* = 6) and compound **h2** (*p* < 0.01, *n* = 6) and **h3** (*p* < 0.001, *n* = 6), while **h1**, **h4** and **h5** showed no prominent (*p* > 0.05, *n* = 6) results (Figure 5A). Similarly, upon administration with scopolamine, BChE level was significantly increased 28.17 ± 1.17 µmol/min/mg of protein (*p* < 0.001, *n* = 6) compared to the normal control group (14.72 ± 3.27 µmol/min/mg of protein), which was significantly reversed (*p* < 0.01, *n* = 6) by pretreatment with donepezil 20.69 ± 1.73 µmol/min/mg of protein and the tested compounds **h3** (*p*< 0.01, *n* = 6) and **h2** (*p* < 0.05, *n* = 6), while **h1**, **h4** and **h5** showed no promising results (*p* > 0.05, *n* = 6) (Figure 5B). 

## 3. Discussion

The five synthesized mono-carbonyl curcumin analogs were screened in an in vitro experiment against two enzymes, AChE and BChE. Compounds **h2** and **h3** showed significantly higher anticholinesterase activity. It has been reported that curcumin maintains acetylcholine level by inhibiting cholinesterases, thus enhancing memory performance [24]. Cholineesterase inhibitors inhibit the cholinesterase enzyme from degrading ACh and enhance their level, as well as the period of nerve impulse transmission [26]. The inhibition of cholinesterases by the synthesized curcumin analogs indicates the cholinesterase inhibitory potential of the curcumin analogs [24].

The anticholinesterase potential of the synthesized curcumin analogs was confirmed and validated through the molecular docking approach using the MOE-Dock protocol. To predict the interaction between AChE and BChE and ligand molecules, the ligX application within the package was used [25]. All of the synthesized curcumin analogs were docked against both targets—AChE and BChE—using the MOE tool, and all compounds showed good binding affinity with the target proteins. The modes of binding of the synthesized curcumin analogs were in correlation with the calculated IC_50_ values, whereby active compound **h3** showed the most promising results in the case of AChE. Compound **h3** interacted with two active site residues (Tyr124 and Gly342). The active site residue Tyr124 interacted with the chlorobenzene moiety, and Gly342 interacted with carbonyl group of the compound. 

Similarly, as observed in the case of AChE, compound **h3** was the most active against BChE, and interacted with two active site residues (Trp82 and Ser198). The active site residue Trp82 interacted with the chlorobenzene moiety and Ser198 interacted with c the arbonyl group of the compound. The chlorobenzene moiety formed a hydrogen bond with Tyr332, and the di-carbonyl group was observed to form a hydrogen bond with Gly116 of the BChE binding site. The current study suggested that the synthesized curcumin analogs inhibited the cholinesterases in the in vitro analysis, and may be used as therapeutic agents for neurodegenerative disorders. 

The elevated plus maze (EPM) model is considered one of the most reliable paradigms for memory evaluation [27]. Cognition in rodents has been extensively examined using EPM. This apparatus work on the principle of rodents’ innate aversion to open and high spaces [28]. EPM is one of the behavioral models that can be used to study brain regions (e.g., hippocampus, limbic regions).]. Behavioral studies were conducted to determine the possible anti-amnesic potential of the synthesized compounds in an EPM mice model. The synthesized curcumin analogs significantly (*p* < 0.001) reduced TLT compared to the amnesic group, indicating that the scopolamine-induced memory impairment (amnesia) had been reversed. These results for the synthesized compounds in the EPM mouse model indicated memory improvement through a reduction in transfer latency in terms of retention session compared to the acquisition session. Earlier administration of the synthesized curcumin analogs attenuated scopolamine-induced memory deficits in mice [29]. The improvement in memory in EPM with the curcumin analogs demonstrates the potential benefits of curcumin. Curcumin significantly enhances learning and memory function and protects brain neurons from degeneration, as has been previously reported [24,30]. These results demonstrate that scopolamine-induced amnesia is associated with impaired learning and memory in behavioral models in mice. The administration of the curcumin analogs to the animals played protective role in learning and memory after the addition of scopolamine.

Novelty is used to better understand how a novel stimulus affects animal behaviors. An animal’s behavior can be changed by novel stimuli, which can also provoke a stress response, and increase plasma corticosterone levels, which is a major stress index, and this provides information on whether retention in a novel environment is fearful and stressful [31]. The behavior of rodents when exploring a previously known stimulus in these behavioral tests is central to animal models of human amnesia [32]. The evaluation of memory on the basis of the novel object recognition test is a very useful technique [33,34]. The anti-amnesic activity of the synthesized curcumin analogs for both short-term memory and long-term memory was evaluated on the basis of the NOR test mouse model. It was observed that the exploration time for the novel object was significantly higher when treated with curcumin analogs in the test phase. Donepezil, as a standard drug, significantly enhanced the exploration time for the novel object compared to the (scopolamine-treated) amnesic group. Compounds **h2** and **h3** significantly enhanced the exploration time for the novel object compared to the scopolamine-treated group. The anti-amnesic potential in of the synthesized compounds terms of long-term memory was evaluated after testing for short-term memory. The increase in % DI of the synthesized compounds showed improvement in learning and memory. The results of this research were consistent with in vitro anticholinesterase potential and were further validated by the molecular docking study, and this was in agreement with the study reported in [3,35].

Cholinesterases degraded the acetylcholine at the synaptic cleft, leading to diminished cholinergic transmission. Compounds that enhance AChE level cause memory impairment by decreasing ACh levels, with scopolamine doing the same in the amnesic group. The standard drug donepezil and the synthesized curcumin analogs restored memory in mice, as was observed in behavioral studies with significantly reduced AChE levels in mouse brain. Acetyl cholinesterase inhibitors increase the level of ACh by inhibiting the AChE enzyme, enhancing both the period and the transmission of nerve impulses [26]. The substantial reduction in AChE and BChE levles and the significant increase in ACh levels in mouse brain achieved by subsequent administration of the synthesized curcumin analogs indicates the potential role of curcumin analogs as anticholinesterases [23,24]. The synthesized curcumin analogs showed cholinesterase inhibitory activity, and these biochemical changes are responsible for neuroprotective and anti-amnesic activity in mice. 

## 4. Materials and Methods

### 4.1. Materials 

All the chemicals and solvents were of analytical grade, obtained from Sigma Aldrich (Merck, Darmstadt, Germany), or purchased from the local market. TLC (thin-layer chromatography) on Merck 60F_254_ silica gel plates was used for the progress of reaction. The ^1^H-NMR spectra (300 MHz) were recorded employing a Bruker Varian Mercury 300 MHz FT Spectrometer (Bruker, Billerica, MA, USA), in CDCl_3_. The structures of some of the compounds were confirmed by ^13^CNMR, FT-IR (Islamabad, Pakistan) and HR-MS (Islamabad, Pakistan) analysis.

### 4.2. Methodology

#### 4.2.1. General Procedure for the Synthesis Curcumin Analogs

Synthesis of mono-carbonyl curcumin analogs was accomplished using substituted aldehydes (4-methylbenzaldehyde 0.235 mL, 4-methoxybenzaldehyde 0.243 mL, 4-chlorobenzaldehyde 0.281 gm, 4-(Dimethylamino)benzaldehyde) 0.298 gm, and 4-nitrobenzaldehyde 0.302 gm) with 0.074 mL of acetone. A mixture of 2 mmol aldehydes was reacted with 1 mmol of the respective ketone in a 2:1 ratio using 15 mL cold ethanol as solvent and adding 40% of 10 mL sodium hydroxide aqueous solution and continuously stirring for 2 h. TLC plates were used for the reaction process. Finally, HCl (50%) solution 10 mL was added to neutralize the catalyst. The filtered dried product was recrystallized in ethyl acetate or ethanol [36]. 

#### 4.2.2. Synthesis of (1*E*,4*E*)-1,5-Di-p-tolylpenta-1,4-dien-3-one (**h1**)

Yield: 67.8%, yellow needle crystals, melting point: 173–176 °C, solubility: chloroform, ethyl acetate, Rf value: 0.80 in ethyl acetate *n*-hexane (3:7) mixture. ^1^H NMR (CDCl_3_, 400 MHz): δ 7.74 (d, *J* = 15.9 Hz, 2H), 7.54 (d, *J* = 8.1 Hz, 4H), 7.30–7.22 (m, 4H), 7.07 (d, *J* = 15.9 Hz, 2H), 2.41 (s, 6H). ^13^CNMR, Mass and FT-IR spectroscopy data were reported by Caroline Carapina et al., 2019 [36].

#### 4.2.3. Synthesis of (1*E*,4*E*)-1,5-bis(4-Methoxyphenyl)penta-1,4-dien-3-one (**h2**)

Yield: 72.5%, pale yellow crystals, melting point: 127–130 °C, solubility: chloroform, ethyl acetate, Rf value: 0.54 in ethyl acetate: *n*-hexane (3:7) mixture. ^1^H NMR (CDCl_3_, 300 MHz): δ 7.72 (d, *J* = 15.9 Hz, 2H), 7.59 (m, 4H), 7.06–6.88 (m, 6H), 3.88 (s, 6H). ^13^CNMR, Mass and FT-IR spectroscopy data were reported by Caroline Carapina et al., 2019 [6].

#### 4.2.4. Synthesis of (1*E*,4*E*)-1,5-bis(4-Chlorophenyl)penta-1,4-dien-3-one (**h3**)

Yield: 47.2%, light yellow crystals, melting point: 183–185 °C, solubility: chloroform, ethyl acetate, Rf value: 0.76 in ethyl acetate: *n*-hexane (3:7) mixture. ^1^H NMR (CDCl_3_, 400 MHz): δ 7.72 (d, *J* = 15.9 Hz, 2H), 7.63–7.57 (m, 4H), 7.02–6.93 (m, 6H). ^13^CNMR, Mass and FT-IR spectroscopy data were reported by Caroline Carapina et al., 2019 [36]. 

#### 4.2.5. Synthesis of (1*E*,4*E*)-1,5-bis(4-(Dimethylamino)phenyl)penta-1,4-dien-3-one (**h4**)

Yield: 85.1%, red crystalline powder, melting point: 185–188 °C, solubility: chloroform, ethyl acetate, Rf value: 0.55 in ethyl acetate: *n*-hexane (3:7) mixture. ^1^H NMR (CDCl_3_, 400 MHz): δ 7.71 (d, *J* = 15.7 Hz, 2H), 7.53 (dd, *J* = 10.1, 7.3 Hz, 4H), 6.91 (d, *J* = 15.7 Hz, 2H), 6.74–6.70 (m, 4H), 3.06 (s, 12H). ^13^CNMR, Mass and FT-IR spectroscopy data were reported by Guang Liang et al., 2008 [19].

#### 4.2.6. Synthesis of (1*E*,4*E*)-1,5-bis(4-Nitrophenyl)penta-1,4-dien-3-one (**h5**)

Yield: 74.3%, brown crystalline powder, melting point: 219–221 °C, solubility: ethyl acetate, Rf value: 0.13 in ethyl acetate: *n*-hexane (3:7) mixture. ^1^H NMR (CDCl_3_, 400 MHz): δ 8.08 (d, *J* = 15.9 Hz, 4H), 8.41(m, 4H), 6.58 (s, 4H). HR-MS: *m*/*z* = 324.29. ^13^CNMR, and FT-IR data were reported by Pankaj Naikwadi et al., 2017 [37].

### 4.3. Molecular Docking Study

#### 4.3.1. Protein Preparation

The three-dimensional structures of AChE and BChE used in current research were taken from PDB (Protein Data Bank IDs: 2gyu and 4tpk). The structures were refined by removing water molecules and performing 3D protonation of the protein molecules. The energy minimization algorithm of the MOE (molecular operating environment) http://www.chemcomp.com/softw (accessed on 5 April 2021) are package was used to carry out energy minimization of the 3D protonated protein molecules. The energy minimization parameters were as follows: 0.05 gradient, MMFF94X + Solvation (force field), current geometry in chiral constraint. When the gradient fell below 0.5 of the root mean square, the energy minimization was terminated by default [38]. The final product (minimized protein structures) was used to simulate molecular docking. 

#### 4.3.2. Ligand Preparation

The compounds used as ligands were retrieved using the MOE Builder application [39]. Like the protein, the energy minimization algorithm of the MOE tool was used to carry out energy minimization of all the ligand molecules. The ligand molecules (minimized) were saved in mol2 format and exported to a single database of .mdb file format. Finally, the prepared database of ligand molecules was subjected to molecular docking via MOE-Dock [40].

#### 4.3.3. Receptor Preparation

Donepezil inhibitor PDB ID: 4EY7 homodimer complex with AChE with a resolution of 2.35 Å and tacrine inhibitor PDB ID: 4BDS monomer complex with human BChE with a resolution of 2.10 Å were used, and for docking implementation, human AChE chain B was selected. The addition of the missing AChE and BChE residues was performed and adjusted under Molecular Operating Environment (MOE). The water molecules were deleted except for those water molecules involved in the interactions. Hydrogen atoms were added to the complex structures, and energy minimization was achieved [38]. 

#### 4.3.4. Re-docking Setup

Re-docking was executed for docking software validation and the MOE software was validated. The co-crystallized ligand in re-docking was incorporated into the AChE and BChE active sites, and the fitness of each re-docked pose was calculated on the basis of RMSD (root-mean-square deviation) [41].

### 4.4. Pharmacological Activities

#### 4.4.1. Anticholinesterase Assay

Evaluation of anticholinesterase activity of the synthesized compounds was performed according to Ellman’s method [38] and was determined by obtaining AChE and BChE enzymes from electric eel and equine serum, respectively. DMSO was used for the dissolution of test samples (1 mg/mL), which were diluted in phosphate buffer (0.1 M) to concentrations from 1000 to 62.5 µg/mL. Phosphate buffer in 0.1 M, pH 8.0 was used for the dilution of 518 U/mg AChE and 7–16 U/mg BChE to obtain final concentrations of 0.03 U/mL and 0.01 U/mL for AChE and BChE, respectively. Substrate solutions of 0.5 mM ATchI, 0.2273 mM DTNB and 0.5 mM BTchI in distilled water were made and maintained separately in Eppendorf tubes at 8 °C in a refrigerator. The assay was started by adding 5 µL enzyme solution to a cuvette, then adding 205 µL test sample followed by 5 µL DTNB reagent. A 5 µL volume of substrate solution was added, and the solution mixture was maintained for 15 min in water bath at 30 °C. A spectrophotometer was used to measure absorbance at 412 nm at 30 °C with a reaction time of 4 min. The experiment was run in triplicate, and galanthamine was used as the positive control. The following formula was used for calculation of % enzyme inhibition.

V = ∆Abs/∆t

Percent enzyme inhibition = 100% enzyme activity

Percent enzyme activity = (100 × V/V_max_)

where V_max_ is the enzyme activity in the absence of inhibitor.

#### 4.4.2. Animals, Dosing and Grouping

Balb/C mice iwth a weight of (17–23 gm) were procured from the NIH (National Institute of Health), Islamabad, which were kept for breeding under favorable conditions in the animal house of the Pharmacy Department, Shaheed Benazir Bhutto University Sheringal Dir (Upper). Light/dark cycle of about 12 h each was maintained, with relative humidity of 50–55% at 22–25 °C. This work was conducted with approval from the Departmental Ethical Committee (SBBU/IEC-20-02) of the Scientific Procedure Issue-I of the University of Malakand in compliance with provision of the 2008 Animal Byelaws.

#### 4.4.3. In Vivo Analysis

The anti-amnesic potential of the synthesized curcumin analogs was determined according to the experimental protocol.

Mice were divided into thirteen groups with 6 mice in each group.

Group I. Normal control group (vehicle treated) received 10 mL/kg (i.p.) normal saline.

Group II. Scopolamine-treated (amnesic) group received 1 mg/kg (i.p.) scopolamine.

Group III: Donepezil-treated (standard) group received 2 mg/kg (p.o.) donepezil.

Group IV–XIII. Sample (curcumin analog)-treated groups. Synthesized curcumin analogs were administered in doses of 7.5 mg/kg and 15 mg/kg (p.o.), respectively.

The groups were kept for 7 days at various treatments doses. At the end of last dose of the treatment period on the 7th day, scopolamine was given to each mouse in a dose of 1 mg/kg (i.p.) 60 min after donepezil or the tested compounds except for group I, and after 30 min, cognitive paradigms were evaluated [28].

##### Elevated Plus Maze Model (EPM)

Determination of memory enhancement potential using EPM mice model is the most reliable paradigm [5]. The EPM consisted of two open arms and two closed arms with dimensions of 16 cm × 5 cm × 12 cm, and was made from two poly acrylic sheets and designed as a plus sign with a central platform with dimensions of 5 cm × 5 cm, which was elevated 25 cm from the floor on a wooden stand. Mice were placed in the open arm in such a position that their direction was facing away from the central platform; then, the transfer latency time (TLT) was noted when the mice moved to the closed arm with its four legs from the open arm and then returned to their home cage. Mice explored the apparatus for 90 s and were gently moved to the closed arm if they failed to find the closed arm in the given time, in which case 90 s was assigned as transfer latency time for that mouse. The initial transfer latency 45 min after scopolamine dose was recorded. The maze was explored by the mice for 10 s, and then they were sent back to their home cage, and TLT (retention of latency) was recorded again 24 h after scopolamine administration. The reduction in TLT showed the memory-enhancing potential of the compounds. The following formula was used for calculation of inflexion ratio (*IR*).

$$IR=\frac{L0-L1}{L0}$$

where *L*1 = initial transfer latency (s) and *L*0 = retention transfer latency (s).

##### Novel Object Recognition Test

The synthesized curcumin analogs were tested for possible memory enhancement potential by using novel object recognition test apparatus, which was designed using plexi glass with dimension of 40 cm × 40 cm × 30 cm [6]. The mice, after acclimatization period, were allowed to move for 2–3 min one day before the test for habituation. In the two corners of the NORT apparatus, two identical objects were placed on the test day in the sample phase (T1), and the object exploration time was recorded. The objects were regarded as being explored when the mice touched the objects or directed their nose toward the object at a distance of less than 2 cm, and 24 h after T1, the test phase (T2) was started, in which one of the familiar objects was replaced with a novel (new) object. Mice again freely explored the objects in similar manner to that in T1, and the exploration time for the novel (new) object and the familiar (F) object were noted separately. The DI (discrimination index) was measured using the following formula:

$$DI=\frac{N-F}{N+F}$$

#### 4.4.4. Isolation of Brain Hippocampus for Biochemical Assessment

The mice were sacrificed immediately after the behavioral study by cervical dislocation to provide quick and painless death to each animal before decapitation. Brain of each animal was excised in phosphate buffer saline (chilled) and incorporated into biochemical biomarker analysis [5].

##### Cholinesterase (AChE and BChE) Activity

Cholinesterase (AChE and BChE) levels were determined in the excised brain hippocampus using Ellman’s method with slight modification [42]. Supernatant (0.4 mL), 0.1 M/L, pH 8 phosphate buffer (2.6 mL), and 100 µL of DTNB (5,5′-dithiobis-2-nitrobenzoic acid) and were added into cuvette and mixed. Spectrophotometer at 412 nm was run and absorbance was measured several times, then 20 mL acetyl thiocholine iodide/butyrylthiocholine chloride was mixed into the reaction as substrate at an interval of 2 min, and variation in absorbance was noted and the changes in absorbance per min were recorded. The following formula was used for calculation of cholinesterase level:

R = 5.74 × 10^−4^ × A/CO

where ‘R’ is the moles (rate) of substrate hydrolyzed/min/mg of protein, (A) is the change in absorbance/min, and ‘CO’ is concentration (20 mg/mL) of protein.

### 4.5. Statistical Analysis

The measured data are presented as mean ± SEM. Data were statistically analyzed using Graph Pad Prisom 5.01. Analysis of variance one-way (ANOVA) and Dunnet’s multiple comparison tests were applied to the data set.

## 5. Conclusions

Five mono-carbonyl curcumin analogs were synthesized and screened for in vitro anticholinesterase activities, validated by molecular docking studies. Based on the in vitro results, the synthesized compounds were further subjected to in vivo and ex vivo analysis. Compounds **h2** and **h3** showed significantly higher memory enhancing effects, as assessed on the basis of in vivo scopolamine-induced memory deficits in mice in EPM and NORT behavioral tests, which was further supported by ex vivo analysis. It is evident from the current study that the synthesized curcumin analogs improved cognition in mice and reduced the progression of dementia. However, further research work is needed to investigate the exact mechanism and cellular pathways involved.

## Figures and Tables

**Table 1:** Compound codes, molecular structures, IC_50_ values, docking scores and binding affinity of the synthesized compounds against AChE and BChE.

Comp.	AChEI (IC_50_)	Docking Score	Binding Energy (GBVI/WSA)	BChEI (IC_50_)	Docking Score	Binding Energy (GBVI/WSA)
h1	104.72	–8.354	–14.164	243.12	–7.874	–13.491
h2	93.27	–10.176	–17.317	147.92	–8.360	–15.082
h3	29.39	–12.412	–21.543	67.35	–11.537	–19.728
h4	294.77	–7.954	–13.674	512.77	–7.612	–14.011
h5	632.37	–7.018	–13.006	988.35	–6.603	–11.225

Note: GBVI/WSA (Generalized-Born Volume Integral/Weighted Surface Area) is a scoring function of the estimated free energy from a given pose of a binding ligand. Lower scores indicate a more favorable pose in all scoring functions. In the molecular docking studies, the choice of active and non-active molecules was based on binding energies and docking scores.

**Table 2:** In vitro cholinesterase inhibition potential of curcumin analogs.

Comp.	Conc. µg/mL	Mean ± SEM	AChEI (IC_50_)	Mean ± SEM	BChEI (IC_50_)
h1	1000	79.67 ± 0.21 ^ns^	104.72	62.16 ± 0.04 ***	243.12
	500	68.07 ± 0.18 ***		56.42 ± 0.16 ***	
	250	58.12 ± 0.16 ***		51.91 ± 0.14 ***	
	125	54.34 ± 0.17 ***		46.25 ± 0.13 ***	
	62.5	45.63 ± 0.13 ***		37.16 ± 0.12 ***	
h2	1000	76.23 ± 0.15 ^ns^	93.27	69.77 ± 0.03 ***	147.92
	500	73.79 ± 0.12 ^ns^		64.38 ± 0.06 ***	
	250	61.21±0.18 ***		57.22 ± 0.01 ***	
	125	53.57 ± 0.17 ***		48.61 ± 0.15 ***	
	62.5	46.88 ± 0.15 ***		41.82 ± 0.18 ***	
h3	1000	83.31 ± 0.13 ^ns^	29.39	78.72 ± 0.16 *	67.35
	500	77.25 ± 0.11 ^ns^		69.52 ± 0.12 ***	
	250	69.51 ± 0.13 ^ns^		62.41 ± 0.19 ***	
	125	62.44 ± 0.16 ^ns^		58.33 ± 0.13 ***	
	62.5	53.53±0.12 *		49.25 ± 0.02 ***	
h4	1000	63.51 ± 0.17 ***	294.77	56.32 ± 0.18 ***	512.77
	500	58.16 ± 0.11 ***		49.51 ± 0.16 ***	
	250	46.32 ± 0.15 ***		42.62 ± 0.12 ***	
	125	39.45 ± 0.04 ***		35.33 ± 0.19 ***	
	62.5	31.41 ± 0.13 ***		27.16 ± 0.11 ***	
h5	1000	58.41 ± 0.13 ***	632.37	51.82 ± 0.16 ***	988.35
	500	47.33 ± 0.16 ***		43.51 ± 0.13 ***	
	250	41.27 ± 0.04 ***		32.73 ± 0.01 ***	
	125	35.51 ± 0.06 ***		20.26 ± 0.31 ***	
	62.5	26.45 ± 0.18 ***		16.22 ± 0.83 ***	
Galanthamine	1000	81.85 ± 0.18	26.58	83.53 ± 0.20	21.30
	500	76.59 ± 0.30		78.62 ± 0.17	
	250	69.75 ± 0.14		73.42 ± 0.11	
	125	64.47 ± 0.49		66.20 ± 0.15	
	62.5	59.12 ± 0.34		61.35 ± 0.18	

All values are presented as mean ± SEM, (*n* = 3). Galanthamine was used as standard control. Significantly different values are with reference to the standard control; *p*-value <0.001, <0.01, <0.05, >0.05 are expressed as ***, **, * and ‘ns’, respectively.

**Table 3:** Effect of synthesized curcumin analogs **h1**–**h5** on alteration behavior and transfer latency in mice.

Samples	Treatment/Dose	Retention (sec)
Control	Normal saline, p.o	36.42 ± 1.81
Scopolamine (Scop)	1 mg/kg, i.p negative control	67.31 ± 1.24 ***
Donepezil (DZP)	2 mg/kg p.o	25.57 ± 1.93 ***
h1	7.5 mg/kg, p.o	33.13 ± 2.16 *
	15 mg/kg, p.o	26.18 ± 2.32 ***
h2	7.5 mg/kg, p.o	31.51 ± 2.73 **
	15 mg/kg, p.o	25.32 ± 2.42 ***
h3	7.5 mg/kg, p.o	25.26 ± 2.51 ***
	15 mg/kg, p.o	21.27 ± 2.92 ***
h4	7.5 mg/kg, p.o	35.71 ± 1.13 *
	15 mg/kg, p.o	30.42 ± 1.24 **
h5	7.5 mg/kg, p.o	34.71 ± 1.37 *
	15 mg/kg, p.o	31.51 ± 1.54 **

All values are presented as mean ± SEM (*n* = 6), *p <* 0.001 vs. normal control. Positive control and tested samples received Scopolamine 1 mg/kg i.p. Significantly different values are with reference to the scopolamine-treated group; *p*-value *<*0.001, *<*0.01, *<*0.05 are expressed as ***, **, * respectively. One-way ANOVA followed by Dunnett’s multiple comparison tests was applied on these data.

## Data Availability

All data contained within this article.

## Sample Availability

Samples of the compounds are available will be provided on reasonable request.
